# Methyl 4′-isobutyl-2,2′-dimethyl-1,3-dioxo-2,3-dihydro-1*H*,4′*H*-spiro­[iso­quinoline-4,5′-oxazole]-4′-carboxyl­ate

**DOI:** 10.1107/S1600536811019003

**Published:** 2011-05-25

**Authors:** Hoong-Kun Fun, Ching Kheng Quah, Kai Xu, Yan Zhang

**Affiliations:** a X-ray Crystallography Unit, School of Physics, Universiti Sains Malaysia, 11800 USM, Penang, Malaysia; b School of Chemistry and Chemical Engineering, Nanjing University, Nanjing, 210093, People’s Republic of China

## Abstract

In the isoquinoline ring system of the title mol­ecule, C_19_H_22_N_2_O_5_, the *N*-heterocyclic ring is in a half-boat conformation. The dioxa-2-aza­spiro ring is essentially planar [maximum deviation = 0.042 (1) Å] and forms a dihedral angle of 81.85 (4)° with the benzene ring. In the crystal, the mol­ecules are linked *via* inter­molecular C—H⋯O hydrogen bonds into chains along [010].

## Related literature

For general background to and the potential biological activity of the title compound, see: Pollers-Wieers *et al.* (1981 ▶); Malamas *et al.* (1994 ▶); Yu *et al.* (2010 ▶); Mitchell *et al.* (1995 ▶, 2000 ▶); Badillo *et al.* (2010 ▶); Wang *et al.* (2010 ▶); Nair *et al.* (2002 ▶); Huang *et al.* (2011 ▶). For the stability of the temperature controller used in the data collection, see: Cosier & Glazer (1986 ▶). For standard bond-length data, see: Allen *et al.* (1987 ▶). For ring conformations, see: Cremer & Pople (1975 ▶). For related structures, see: Fun *et al.* (2011*a* ▶,*b* ▶,*c* ▶,*d* ▶).
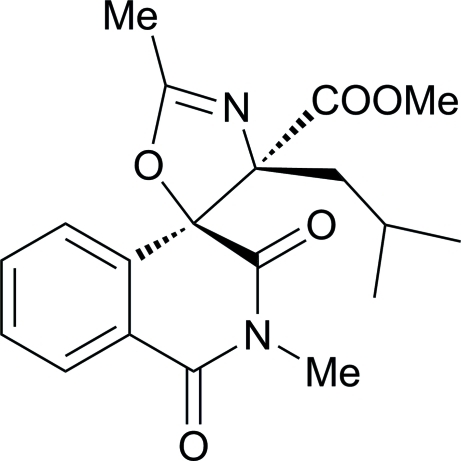


## Experimental

### 

#### Crystal data

C_19_H_22_N_2_O_5_
*M* *_r_* = 358.39
Triclinic, 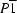

*a* = 8.6764 (1) Å
*b* = 8.9366 (1) Å
*c* = 12.0684 (2) Å
α = 93.495 (1)°
β = 109.892 (1)°
γ = 98.426 (1)°
*V* = 864.22 (2) Å^3^
*Z* = 2
Mo *K*α radiation
μ = 0.10 mm^−1^
*T* = 100 K
0.40 × 0.29 × 0.18 mm

#### Data collection

Bruker SMART APEXII CCD area-detector diffractometer
Absorption correction: multi-scan (*SADABS*; Bruker, 2009 ▶) *T*_min_ = 0.961, *T*_max_ = 0.982
25638 measured reflections
6231 independent reflections
5381 reflections with *I* > 2σ(*I*)
*R*_int_ = 0.027

#### Refinement

*R*[*F* ^2^ > 2σ(*F* ^2^)] = 0.039
*wR*(*F* ^2^) = 0.113
*S* = 1.05
6231 reflections
240 parameters
H-atom parameters constrained
Δρ_max_ = 0.46 e Å^−3^
Δρ_min_ = −0.24 e Å^−3^

### 

Data collection: *APEX2* (Bruker, 2009 ▶); cell refinement: *SAINT* (Bruker, 2009 ▶); data reduction: *SAINT*; program(s) used to solve structure: *SHELXTL* (Sheldrick, 2008 ▶); program(s) used to refine structure: *SHELXTL*; molecular graphics: *SHELXTL*; software used to prepare material for publication: *SHELXTL* and *PLATON* (Spek, 2009 ▶).

## Supplementary Material

Crystal structure: contains datablocks global, I. DOI: 10.1107/S1600536811019003/rz2597sup1.cif

Structure factors: contains datablocks I. DOI: 10.1107/S1600536811019003/rz2597Isup2.hkl

Supplementary material file. DOI: 10.1107/S1600536811019003/rz2597Isup3.cml

Additional supplementary materials:  crystallographic information; 3D view; checkCIF report

## Figures and Tables

**Table 1:** Hydrogen-bond geometry (Å, °)

*D*—H⋯*A*	*D*—H	H⋯*A*	*D*⋯*A*	*D*—H⋯*A*
C2—H2*A*⋯O4^i^	0.93	2.56	3.4353 (11)	158
C4—H4*A*⋯O2^ii^	0.93	2.56	3.2878 (11)	136
C12—H12*C*⋯O4^i^	0.96	2.59	3.3600 (11)	138

Symmetry codes: (i) 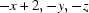; (ii) 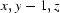.

**Fig. 1.**
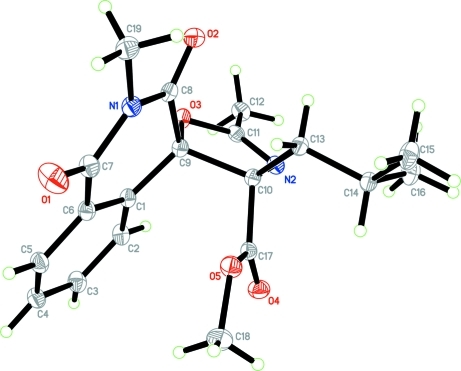
The molecular structure of the title compound showing 50% probability displacement ellipsoids for non-H atoms.

**Fig. 2.**
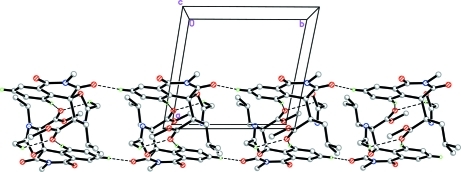
The crystal structure of the title compound, viewed along the c axis. H atoms not involved in hydrogen bonds (dashed lines) have been omitted for clarity.

## supplementary crystallographic information

### Comment

Isoquinolines are often found in bioactive natural products. They have been used to build blocks of benzo[c]phenanthridine alkaloids. (Pollers-Wieers *et al.*, 1981; Malamas *et al.*, 1994; Yu *et al.*, 2010). Isoquinoline-1,3,4-trione and its derivatives have been reported to be redox mediators of photosystems I and have been used as herbicides (Mitchell *et al.*, 2000; 1995). Oxazole rings are also found in some bioactive natural products such as Annuloline and Ostreogrycin A. Spirooxazoles have emerged as attractive synthetic targets because of their potent bioactivity (Badillo *et al.*, 2011; Wang *et al.*; 2010; Nair *et al.*, 2002). The title compound, which was derived from isoquinoline-1,3,4-trione and oxazoles (Huang *et al.*, 2011), may has a potential use in biochemical and pharmaceutical fields. Due to the importance of the isoquinoline-1,3,4-trione derivatives, we report in this paper the crystal structure of the title compound with a relative configuration of (4S^*^, 4'S^*^).

In the title racemic compound, Fig. 1, atoms C9 and C10 are the stereo centers. The isoquinoline ring system (N1/C1-C9) is not completely planar, the *N*-heterocyclic ring (N1/C1/C6-C9) being distorted towards a half-boat conformation with atom C9 deviating by 0.167 (1) Å from the mean plane through the remaining atoms, puckering parameters (Cremer & Pople, 1975) Q = 0.2505 (9) Å, Θ =  114.3 (2)° and φ = 73.8 (2)°. The dioxa-2-azaspiro ring (N2/O3/C9-C11) is essentially planar [maximum deviation of 0.042 (1) Å at atoms C9 and C10] and it inclines at a dihedral angle of  81.85 (4)° with the benzene ring (C1-C6). Bond lengths (Allen *et al.*, 1987) and angles are within normal ranges and comparable to related structures (Fun *et al.*, 2011*a*,*b*,*c*,*d*).

In the crystal packing (Fig. 2), the molecules are linked *via* intermolecular C2–H2A···O4, C12–H12C···O4 and C4–H4A···O2 hydrogen bonds (Table 1) into one-dimensional chains along [010].

### Experimental

The title compound was the main product from the acid-catalyzed transformation of the photocycloadduct of isoquinoline-1,3,4-trione and 4-isobutyl-5-methoxy-2-methyloxazole. The compound was purified by flash column chromatography with ethyl acetate/petroleum ether (1:4 *v*/*v*) as eluents. X-ray quality crystals of the title compound were obtained from slow evaporation of an acetone/petroleum ether solution (1:5 *v*/*v*). M.p. 421-423 K.

### Refinement

All H atoms were positioned geometrically and refined using a riding model with C–H = 0.93 - 0.97 Å and *U*_iso_(H) = 1.2 or 1.5 *U*_eq_(C). A rotating-group model was applied for the methyl groups. The highest residual electron density peak is located at 0.75 Å from C17 and the deepest hole is located at 0.53 Å from C11.

### Crystal data

**Table:**

C_19_H_22_N_2_O_5_	*Z* = 2
*M**_r_* = 358.39	*F*(000) = 380
Triclinic, *P*1	*D*_x_ = 1.377 Mg m^−^^3^
Hall symbol: -P 1	Mo *K*α radiation, λ = 0.71073 Å
*a* = 8.6764 (1) Å	Cell parameters from 9993 reflections
*b* = 8.9366 (1) Å	θ = 2.3–35.1°
*c* = 12.0684 (2) Å	µ = 0.10 mm^−^^1^
α = 93.495 (1)°	*T* = 100 K
β = 109.892 (1)°	Block, colourless
γ = 98.426 (1)°	0.40 × 0.29 × 0.18 mm
*V* = 864.22 (2) Å^3^	

### Data collection

**Table:**

Bruker SMART APEXII CCD area-detector diffractometer	6231 independent reflections
Radiation source: fine-focus sealed tube	5381 reflections with *I* > 2σ(*I*)
graphite	*R*_int_ = 0.027
φ and ω scans	θ_max_ = 32.5°, θ_min_ = 1.8°
Absorption correction: multi-scan (*SADABS*; Bruker, 2009)	*h* = −13→13
*T*_min_ = 0.961, *T*_max_ = 0.982	*k* = −13→13
25638 measured reflections	*l* = −16→18

### Refinement

**Table:**

Refinement on *F*^2^	Primary atom site location: structure-invariant direct methods
Least-squares matrix: full	Secondary atom site location: difference Fourier map
*R*[*F*^2^ > 2σ(*F*^2^)] = 0.039	Hydrogen site location: inferred from neighbouring sites
*wR*(*F*^2^) = 0.113	H-atom parameters constrained
*S* = 1.05	*w* = 1/[σ^2^(*F*_o_^2^) + (0.0637*P*)^2^ + 0.1577*P*] where *P* = (*F*_o_^2^ + 2*F*_c_^2^)/3
6231 reflections	(Δ/σ)_max_ = 0.001
240 parameters	Δρ_max_ = 0.46 e Å^−^^3^
0 restraints	Δρ_min_ = −0.24 e Å^−^^3^

### Special details

**Table:**

Experimental. The crystal was placed in the cold stream of an Oxford Cryosystems Cobra open-flow nitrogen cryostat (Cosier & Glazer, 1986) operating at 100.0 (1) K.
Geometry. All esds (except the esd in the dihedral angle between two l.s. planes) are estimated using the full covariance matrix. The cell esds are taken into account individually in the estimation of esds in distances, angles and torsion angles; correlations between esds in cell parameters are only used when they are defined by crystal symmetry. An approximate (isotropic) treatment of cell esds is used for estimating esds involving l.s. planes.
Refinement. Refinement of F^2^ against ALL reflections. The weighted R-factor wR and goodness of fit S are based on F^2^, conventional R-factors R are based on F, with F set to zero for negative F^2^. The threshold expression of F^2^ > 2sigma(F^2^) is used only for calculating R-factors(gt) etc. and is not relevant to the choice of reflections for refinement. R-factors based on F^2^ are statistically about twice as large as those based on F, and R- factors based on ALL data will be even larger.

### Fractional atomic coordinates and isotropic or equivalent isotropic displacement parameters (Å^2^)

**Table:**

	*x*	*y*	*z*	*U*_iso_*/*U*_eq_	
O1	0.56369 (10)	−0.13923 (8)	0.36689 (7)	0.02808 (16)	
O2	0.61673 (8)	0.30662 (7)	0.21139 (6)	0.01906 (13)	
O3	0.70178 (7)	0.17637 (7)	0.03819 (5)	0.01584 (12)	
O4	1.13223 (8)	0.01941 (7)	0.19476 (6)	0.02001 (13)	
O5	1.02241 (8)	0.00373 (7)	0.33903 (6)	0.01843 (13)	
N1	0.60176 (9)	0.08767 (8)	0.29574 (6)	0.01547 (13)	
N2	0.98015 (8)	0.26379 (8)	0.11623 (6)	0.01398 (13)	
C1	0.71382 (9)	−0.05589 (9)	0.12763 (7)	0.01375 (14)	
C2	0.75196 (10)	−0.12948 (9)	0.03755 (7)	0.01669 (15)	
H2A	0.7913	−0.0729	−0.0126	0.020*	
C3	0.73110 (11)	−0.28759 (10)	0.02283 (8)	0.01903 (16)	
H3A	0.7556	−0.3364	−0.0378	0.023*	
C4	0.67396 (11)	−0.37359 (10)	0.09800 (8)	0.01860 (16)	
H4A	0.6621	−0.4792	0.0884	0.022*	
C5	0.63485 (10)	−0.30090 (9)	0.18736 (7)	0.01695 (15)	
H5A	0.5961	−0.3578	0.2376	0.020*	
C6	0.65376 (10)	−0.14210 (9)	0.20173 (7)	0.01444 (14)	
C7	0.60524 (10)	−0.06867 (10)	0.29540 (7)	0.01696 (15)	
C8	0.64684 (9)	0.17845 (9)	0.21858 (7)	0.01427 (14)	
C9	0.74719 (9)	0.11548 (9)	0.15004 (7)	0.01297 (13)	
C10	0.94372 (9)	0.18962 (9)	0.21130 (7)	0.01221 (13)	
C11	0.84438 (10)	0.25402 (9)	0.02944 (7)	0.01396 (14)	
C12	0.81958 (11)	0.31809 (10)	−0.08488 (7)	0.01760 (15)	
H12A	0.9226	0.3774	−0.0823	0.026*	
H12B	0.7366	0.3818	−0.0976	0.026*	
H12C	0.7834	0.2365	−0.1486	0.026*	
C13	0.98781 (9)	0.30600 (9)	0.32243 (7)	0.01391 (14)	
H13A	0.9391	0.3950	0.2979	0.017*	
H13B	0.9362	0.2612	0.3753	0.017*	
C14	1.17540 (9)	0.35881 (9)	0.39278 (7)	0.01424 (14)	
H14A	1.2278	0.2682	0.4053	0.017*	
C15	1.19883 (11)	0.43954 (11)	0.51412 (8)	0.02220 (17)	
H15A	1.3156	0.4677	0.5597	0.033*	
H15B	1.1464	0.3723	0.5551	0.033*	
H15C	1.1492	0.5293	0.5039	0.033*	
C16	1.26352 (11)	0.46363 (11)	0.32908 (8)	0.02157 (17)	
H16A	1.3776	0.4980	0.3794	0.032*	
H16B	1.2091	0.5499	0.3110	0.032*	
H16C	1.2593	0.4089	0.2568	0.032*	
C17	1.04488 (9)	0.06210 (9)	0.24456 (7)	0.01415 (14)	
C18	1.10003 (13)	−0.12653 (12)	0.37361 (10)	0.0282 (2)	
H18A	1.0943	−0.1492	0.4490	0.042*	
H18B	1.2145	−0.1044	0.3798	0.042*	
H18C	1.0429	−0.2127	0.3151	0.042*	
C19	0.52591 (11)	0.15905 (10)	0.37250 (8)	0.02020 (16)	
H19A	0.6016	0.2477	0.4198	0.030*	
H19B	0.5024	0.0878	0.4235	0.030*	
H19C	0.4243	0.1883	0.3243	0.030*	

### Atomic displacement parameters (Å^2^)

**Table:**

	*U*^11^	*U*^22^	*U*^33^	*U*^12^	*U*^13^	*U*^23^
O1	0.0440 (4)	0.0228 (3)	0.0276 (4)	0.0051 (3)	0.0250 (3)	0.0085 (3)
O2	0.0179 (3)	0.0170 (3)	0.0230 (3)	0.0048 (2)	0.0072 (2)	0.0034 (2)
O3	0.0145 (2)	0.0189 (3)	0.0129 (2)	0.0007 (2)	0.0035 (2)	0.0058 (2)
O4	0.0199 (3)	0.0196 (3)	0.0222 (3)	0.0068 (2)	0.0083 (2)	0.0017 (2)
O5	0.0200 (3)	0.0176 (3)	0.0172 (3)	0.0030 (2)	0.0053 (2)	0.0072 (2)
N1	0.0155 (3)	0.0164 (3)	0.0166 (3)	0.0018 (2)	0.0087 (2)	0.0016 (2)
N2	0.0163 (3)	0.0142 (3)	0.0129 (3)	0.0018 (2)	0.0071 (2)	0.0031 (2)
C1	0.0132 (3)	0.0142 (3)	0.0124 (3)	0.0001 (2)	0.0037 (2)	0.0014 (2)
C2	0.0182 (3)	0.0171 (3)	0.0141 (3)	−0.0007 (3)	0.0067 (3)	0.0000 (3)
C3	0.0200 (4)	0.0180 (4)	0.0177 (4)	0.0003 (3)	0.0070 (3)	−0.0026 (3)
C4	0.0186 (4)	0.0141 (3)	0.0204 (4)	0.0007 (3)	0.0047 (3)	0.0002 (3)
C5	0.0171 (3)	0.0153 (3)	0.0170 (3)	0.0005 (3)	0.0048 (3)	0.0039 (3)
C6	0.0141 (3)	0.0152 (3)	0.0133 (3)	0.0007 (3)	0.0045 (3)	0.0021 (3)
C7	0.0185 (3)	0.0176 (4)	0.0161 (3)	0.0015 (3)	0.0083 (3)	0.0031 (3)
C8	0.0115 (3)	0.0163 (3)	0.0144 (3)	0.0009 (2)	0.0044 (2)	0.0022 (3)
C9	0.0130 (3)	0.0140 (3)	0.0115 (3)	0.0010 (2)	0.0042 (2)	0.0030 (2)
C10	0.0122 (3)	0.0130 (3)	0.0117 (3)	0.0016 (2)	0.0048 (2)	0.0021 (2)
C11	0.0173 (3)	0.0121 (3)	0.0136 (3)	0.0020 (3)	0.0071 (3)	0.0022 (2)
C12	0.0227 (4)	0.0172 (3)	0.0137 (3)	0.0033 (3)	0.0070 (3)	0.0048 (3)
C13	0.0127 (3)	0.0157 (3)	0.0129 (3)	0.0015 (2)	0.0048 (2)	−0.0007 (3)
C14	0.0126 (3)	0.0153 (3)	0.0140 (3)	0.0012 (2)	0.0044 (2)	0.0002 (3)
C15	0.0170 (4)	0.0295 (4)	0.0163 (4)	−0.0015 (3)	0.0046 (3)	−0.0045 (3)
C16	0.0175 (4)	0.0252 (4)	0.0199 (4)	−0.0029 (3)	0.0065 (3)	0.0034 (3)
C17	0.0137 (3)	0.0130 (3)	0.0135 (3)	0.0005 (2)	0.0028 (3)	0.0014 (2)
C18	0.0264 (4)	0.0233 (4)	0.0332 (5)	0.0066 (4)	0.0054 (4)	0.0157 (4)
C19	0.0194 (4)	0.0222 (4)	0.0224 (4)	0.0029 (3)	0.0126 (3)	−0.0004 (3)

### Geometric parameters (Å, °)

**Table:**

O1—C7	1.2178 (10)	C9—C10	1.6262 (11)
O2—C8	1.2133 (10)	C10—C17	1.5295 (11)
O3—C11	1.3675 (10)	C10—C13	1.5462 (11)
O3—C9	1.4365 (9)	C11—C12	1.4859 (11)
O4—C17	1.2033 (10)	C12—H12A	0.9600
O5—C17	1.3431 (10)	C12—H12B	0.9600
O5—C18	1.4424 (11)	C12—H12C	0.9600
N1—C8	1.3875 (10)	C13—C14	1.5422 (11)
N1—C7	1.4016 (11)	C13—H13A	0.9700
N1—C19	1.4711 (11)	C13—H13B	0.9700
N2—C11	1.2694 (10)	C14—C16	1.5268 (12)
N2—C10	1.4608 (10)	C14—C15	1.5270 (12)
C1—C2	1.3948 (11)	C14—H14A	0.9800
C1—C6	1.3958 (11)	C15—H15A	0.9600
C1—C9	1.5062 (11)	C15—H15B	0.9600
C2—C3	1.3904 (12)	C15—H15C	0.9600
C2—H2A	0.9300	C16—H16A	0.9600
C3—C4	1.3925 (12)	C16—H16B	0.9600
C3—H3A	0.9300	C16—H16C	0.9600
C4—C5	1.3886 (12)	C18—H18A	0.9600
C4—H4A	0.9300	C18—H18B	0.9600
C5—C6	1.3976 (11)	C18—H18C	0.9600
C5—H5A	0.9300	C19—H19A	0.9600
C6—C7	1.4833 (12)	C19—H19B	0.9600
C8—C9	1.5284 (11)	C19—H19C	0.9600
			
C11—O3—C9	107.44 (6)	C11—C12—H12A	109.5
C17—O5—C18	115.39 (7)	C11—C12—H12B	109.5
C8—N1—C7	124.82 (7)	H12A—C12—H12B	109.5
C8—N1—C19	116.23 (7)	C11—C12—H12C	109.5
C7—N1—C19	118.50 (7)	H12A—C12—H12C	109.5
C11—N2—C10	108.20 (6)	H12B—C12—H12C	109.5
C2—C1—C6	119.53 (7)	C14—C13—C10	115.65 (6)
C2—C1—C9	120.39 (7)	C14—C13—H13A	108.4
C6—C1—C9	119.97 (7)	C10—C13—H13A	108.4
C3—C2—C1	119.87 (8)	C14—C13—H13B	108.4
C3—C2—H2A	120.1	C10—C13—H13B	108.4
C1—C2—H2A	120.1	H13A—C13—H13B	107.4
C2—C3—C4	120.66 (8)	C16—C14—C15	109.57 (7)
C2—C3—H3A	119.7	C16—C14—C13	113.42 (7)
C4—C3—H3A	119.7	C15—C14—C13	109.33 (7)
C5—C4—C3	119.68 (8)	C16—C14—H14A	108.1
C5—C4—H4A	120.2	C15—C14—H14A	108.1
C3—C4—H4A	120.2	C13—C14—H14A	108.1
C4—C5—C6	119.92 (8)	C14—C15—H15A	109.5
C4—C5—H5A	120.0	C14—C15—H15B	109.5
C6—C5—H5A	120.0	H15A—C15—H15B	109.5
C1—C6—C5	120.33 (8)	C14—C15—H15C	109.5
C1—C6—C7	121.22 (7)	H15A—C15—H15C	109.5
C5—C6—C7	118.43 (7)	H15B—C15—H15C	109.5
O1—C7—N1	120.23 (8)	C14—C16—H16A	109.5
O1—C7—C6	122.86 (8)	C14—C16—H16B	109.5
N1—C7—C6	116.85 (7)	H16A—C16—H16B	109.5
O2—C8—N1	121.17 (8)	C14—C16—H16C	109.5
O2—C8—C9	121.21 (7)	H16A—C16—H16C	109.5
N1—C8—C9	117.46 (7)	H16B—C16—H16C	109.5
O3—C9—C1	109.04 (6)	O4—C17—O5	124.41 (7)
O3—C9—C8	106.82 (6)	O4—C17—C10	125.85 (7)
C1—C9—C8	113.48 (6)	O5—C17—C10	109.73 (7)
O3—C9—C10	102.23 (6)	O5—C18—H18A	109.5
C1—C9—C10	113.79 (6)	O5—C18—H18B	109.5
C8—C9—C10	110.63 (6)	H18A—C18—H18B	109.5
N2—C10—C17	110.09 (6)	O5—C18—H18C	109.5
N2—C10—C13	110.48 (6)	H18A—C18—H18C	109.5
C17—C10—C13	108.90 (6)	H18B—C18—H18C	109.5
N2—C10—C9	103.04 (6)	N1—C19—H19A	109.5
C17—C10—C9	109.24 (6)	N1—C19—H19B	109.5
C13—C10—C9	114.95 (6)	H19A—C19—H19B	109.5
N2—C11—O3	118.54 (7)	N1—C19—H19C	109.5
N2—C11—C12	127.35 (7)	H19A—C19—H19C	109.5
O3—C11—C12	114.11 (7)	H19B—C19—H19C	109.5
			
C6—C1—C2—C3	−0.47 (12)	O2—C8—C9—C1	−156.00 (7)
C9—C1—C2—C3	175.68 (7)	N1—C8—C9—C1	28.49 (9)
C1—C2—C3—C4	−0.65 (13)	O2—C8—C9—C10	74.70 (9)
C2—C3—C4—C5	1.05 (13)	N1—C8—C9—C10	−100.81 (8)
C3—C4—C5—C6	−0.31 (12)	C11—N2—C10—C17	122.44 (7)
C2—C1—C6—C5	1.20 (12)	C11—N2—C10—C13	−117.25 (7)
C9—C1—C6—C5	−174.97 (7)	C11—N2—C10—C9	6.03 (8)
C2—C1—C6—C7	−177.48 (7)	O3—C9—C10—N2	−7.21 (7)
C9—C1—C6—C7	6.35 (11)	C1—C9—C10—N2	110.20 (7)
C4—C5—C6—C1	−0.81 (12)	C8—C9—C10—N2	−120.67 (7)
C4—C5—C6—C7	177.91 (7)	O3—C9—C10—C17	−124.22 (6)
C8—N1—C7—O1	179.82 (8)	C1—C9—C10—C17	−6.82 (9)
C19—N1—C7—O1	−8.22 (12)	C8—C9—C10—C17	122.32 (7)
C8—N1—C7—C6	−2.85 (12)	O3—C9—C10—C13	113.04 (7)
C19—N1—C7—C6	169.11 (7)	C1—C9—C10—C13	−129.55 (7)
C1—C6—C7—O1	−174.64 (8)	C8—C9—C10—C13	−0.41 (9)
C5—C6—C7—O1	6.66 (13)	C10—N2—C11—O3	−2.63 (10)
C1—C6—C7—N1	8.11 (11)	C10—N2—C11—C12	177.58 (8)
C5—C6—C7—N1	−170.60 (7)	C9—O3—C11—N2	−2.67 (10)
C7—N1—C8—O2	168.43 (8)	C9—O3—C11—C12	177.15 (7)
C19—N1—C8—O2	−3.69 (11)	N2—C10—C13—C14	−72.97 (8)
C7—N1—C8—C9	−16.05 (11)	C17—C10—C13—C14	48.05 (9)
C19—N1—C8—C9	171.82 (7)	C9—C10—C13—C14	170.96 (6)
C11—O3—C9—C1	−114.79 (7)	C10—C13—C14—C16	71.60 (9)
C11—O3—C9—C8	122.21 (7)	C10—C13—C14—C15	−165.79 (7)
C11—O3—C9—C10	5.96 (8)	C18—O5—C17—O4	−5.16 (12)
C2—C1—C9—O3	40.97 (9)	C18—O5—C17—C10	174.99 (7)
C6—C1—C9—O3	−142.90 (7)	N2—C10—C17—O4	−5.43 (11)
C2—C1—C9—C8	159.89 (7)	C13—C10—C17—O4	−126.69 (8)
C6—C1—C9—C8	−23.97 (10)	C9—C10—C17—O4	107.03 (9)
C2—C1—C9—C10	−72.42 (9)	N2—C10—C17—O5	174.42 (6)
C6—C1—C9—C10	103.71 (8)	C13—C10—C17—O5	53.16 (8)
O2—C8—C9—O3	−35.81 (10)	C9—C10—C17—O5	−73.12 (8)
N1—C8—C9—O3	148.68 (7)		

### Hydrogen-bond geometry (Å, °)

**Table:**

*D*—H···*A*	*D*—H	H···*A*	*D*···*A*	*D*—H···*A*
C2—H2A···O4^i^	0.93	2.56	3.4353 (11)	158
C4—H4A···O2^ii^	0.93	2.56	3.2878 (11)	136
C12—H12C···O4^i^	0.96	2.59	3.3600 (11)	138

Symmetry codes: (i) −*x*+2, −*y*, −*z*; (ii) *x*, *y*−1, *z*.
